# Health Tract, No. 78

**Published:** 1865

**Authors:** 


### VACCINATION.

In round numbers, and familiar fractions, of 70,000 Prussian soldiers vaccinated, or re-vaccinated, during 1860, 50,000 were successful—namely, "took." Out of this whole number there was not a single case of small-pox, and only one of varioloid, showing what a perfect protection against small-pox effectual vaccination is; but as three out of four "took" after having been re-vaccinated, there is reason to believe that these might have taken varioloid or small-pox if they had been very directly exposed to it. As confinement to the house in winter makes "catching" diseases more dangerous, and as the virtue of the vaccination of childhood and infancy seems to be exhausted in many cases at puberty, parents who are wise will therefore promptly have every child vaccinated the second time on entering the fourteenth year, especially as it causes very little constitutional disturbance. The family physician should be applied to to use every effort to secure healthy vaccine matter. It would be humanity to make it an indispensable condition of admission into a public school to have a distinct vaccine-mark on all under fourteen, and a certificate of re-vaccination as to all who have entered their fourteenth year.

Vaccination of infants, within a few days after birth, has been attended with accidents more or less serious, and sometimes fatal; and as small-pox is very rare in children under six months of age, it is best, in the case of private families, to defer the operation until the third month, except as to children in hospitals, or in other particularly exposed circumstances. Special efforts should be used to secure proper vaccine matter.

1. Take the lymph from a child not less than five months old.
2. The child's parents should be healthy.
3. The lymph should be taken previous to the ninth day of the existence of the vesicle.
4. Take no blood with the matter.
5. Never vaccinate over a dozen with the same supply, for fear it may have been from a diseased subject.

